# Yeast Trk1 Potassium Transporter Gradually Changes Its Affinity in Response to Both External and Internal Signals

**DOI:** 10.3390/jof8050432

**Published:** 2022-04-22

**Authors:** Jakub Masaryk, Hana Sychrová

**Affiliations:** Laboratory of Membrane Transport, Institute of Physiology, Czech Academy of Sciences, 142 20 Prague, Czech Republic; jakub.masaryk@fgu.cas.cz

**Keywords:** cation homeostasis, *Saccharomyces cerevisiae*, potassium uptake, membrane potential

## Abstract

Yeasts need a high intracellular concentration of potassium to grow. The main K^+^ uptake system in *Saccharomyces cerevisiae* is the Trk1 transporter, a complex protein with four MPM helical membrane motifs. Trk1 has been shown to exist in low- or high-affinity modes, which reflect the availability of potassium in the environment. However, when and how the affinity changes, and whether the potassium availability is the only signal for the affinity switch, remains unknown. Here, we characterize the Trk1 kinetic parameters under various conditions and find that Trk1’s K_T_ and V_max_ change gradually. This gliding adjustment is rapid and precisely reflects the changes in the intracellular potassium content and membrane potential. A detailed characterization of the specific mutations in the P-helices of the MPM segments reveals that the presence of proline in the P-helix of the second and third MPM domain (F820P and L949P) does not affect the function of Trk1 in general, but rather specifically prevents the transporter’s transition to a high-affinity state. The analogous mutations in the two remaining MPM domains (L81P and L1115P) result in a mislocalized and inactive protein, highlighting the importance of the first and fourth P-helices in proper Trk1 folding and activity at the plasma membrane.

## 1. Introduction

A sufficient amount of intracellular potassium is a key prerequisite for yeast survival and growth. Due to its essential role in many cell processes, yeast cells accumulate potassium at an intracellular concentration of 200–300 mM, the highest of all cellular ions [1]. Since yeast cells are able to grow in a wide range of concentrations of external potassium (from a few µM to more than 2.5 M), it is highly important that the capacity for the import and export of potassium is precisely adjusted, to maintain the internal potassium levels within optimal ranges. At their plasma membrane, yeast species employ an array of potassium uptake systems, differing in their structure and mode of action (uniporters Trk, K^+^-H^+^ symporter Hak1 and ATPase Acu1). Based on the genome sequencing of various yeasts, Trk uniporters seem to be the only transporters that exist in all species [2].

The genome of *S. cerevisiae* encodes two potassium transporters, Trk1 and Trk2. The gene encoding the Trk1 protein was first characterized by Gaber et al. [3]. Even though *TRK1* is a non-essential gene, its deletion leads to a sharp increase in potassium requirements and renders cells unable to grow in a low external potassium concentration. Therefore, Trk1 is considered the key player in potassium uptake in *S. cerevisiae*, and the highly similar Trk2 only plays a marginal role in potassium acquisition [4,5]. Trk1 is an integral plasma membrane protein consisting of 1235 amino acids and, based on its primary structure and function, it is ranked among the superfamily of K^+^ transporters (SKT proteins), a conserved group of potassium and sodium transporters in yeast, bacteria and plants [6,7]. Trk1 is specific for potassium and rubidium; nevertheless, it has been shown that some sodium ions may enter via the Trk1 system, if the ratio between potassium and sodium cations in the environment is higher than 1:700 [8]. Moreover, although Trk1 transports cations as a monomer, the protein is also believed to form tetramers in the yeast plasma membrane, and to mediate the efflux of chloride anions via the central pore of the tetramer [9].

While the structure of Trk1 remains experimentally unresolved, several Trk1 models based on the structures of KcsA potassium channel and other members of the SKT-proteins are available [10,11]. According to these models, Trk1 consists of four membrane-pore-membrane (MPM) motifs, each MPM motif includes two transmembrane helices connected by a short pore helix (P-helix), cf. Figure S1. The four MPM motifs are presumably assembled around the central axis, thus, creating a pore for the transport of potassium. In such an arrangement, P-helices are responsible for the structural integrity of the pore region and selectivity filter [11,12].

The most striking feature of Trk1 is the ability to modify its affinity for potassium according to the external availability of this cation. The potential of *S. cerevisiae* cells to quickly modify their capacity for potassium uptake has been known for many years [13]. This feature was assigned to Trk1 shortly after its identification as a key player in potassium uptake [3]. The original ‘dual affinity’ concept presumed a switching between low and high-affinity states as a reaction to transfer from an environment with high potassium concentration to an environment with severe potassium limitations [14]. Though the dominant regulatory role has been always attributed to the external potassium concentration, other regulatory signals, such as the depletion of internal potassium [15] or excess of external sodium [16], have also been suggested to play a role in Trk1’s switching to a high-affinity state. Nonetheless, there is a considerable lack of knowledge beyond these simple concepts, regarding the precise mechanism and regulation of the affinity switch of the Trk1 protein.

So far, only one amino-acid residue has been suggested to play a significant role in the switching of Trk1 to its high-affinity state. Identified by random mutagenesis, the substitution L949P led to an incomplete increase in the affinity, as a response to the potassium limitations [17]. According to the latest Trk1 model [11], Leu949 is localized to the third P-helix. Neither additional data from follow-up experiments, nor any further information about the actual effect of the L949P substitution on Trk1’s performance is available.

Due to the high internal concentration of potassium maintained in yeast cells, in most environments, potassium is imported against a concentration gradient and the uptake is thought to be energized by the plasma-membrane potential [18]. P-type H^+^-ATPase Pma1 is responsible for the extrusion of protons; thus, it plays a crucial role in the regulation of the internal pH and the creation of membrane potential [19]. Apart from enabling the energization of potassium uptake, additional levels of functional connection between Pma1 and Trk1 have been reported [20,21,22,23]. Their nature may be summarized as an interdependence of Pma1 and Trk1 activities and, more specifically, as a need to balance the export of protons by importing potassium and vice versa to maintain an appropriate membrane potential. The precise nature of the signal leading to the parallel increase or attenuation of the activities of Pma1 and Trk1 is currently unknown.

This study focuses on Trk1’s ability to modify its affinity in accordance with the requirements of cells for potassium acquisition. As mentioned above, the basic knowledge of Trk1 existing in two affinity modes has been known for many years. However, it is almost impossible to extract a single concept of a mechanism of the changes in Trk1 affinity from all the published data, as strains with different genetic backgrounds and with various combinations of mutations were used in previous studies, as well as multiple types of growth media or substrates (K^+^, Rb^+^) and diverse experimental procedures. We, therefore, decided to focus our study on a detailed characterization of the changes in the affinity of Trk1 and its regulation and, more specifically, the time course of these changes, the role of external and internal potassium concentrations, as well as the possible involvement of membrane potential. Additionally, in order to unravel the effective mechanism of the affinity changes, we characterized in more detail the effect of L949P and analogous substitutions on the functionality of Trk1.

## 2. Materials and Methods

### 2.1. Strains and Growth Conditions

All strains used in this study are listed in Table S1. The yeast cultures were routinely grown at 30 °C in a standard YNB (0.67% YNB, 2% glucose) or a YNB-F (0.17% YNB-F, 2% glucose, 0.4% ammonium sulphate), which only contained 15 μM K^+^. The desired amount of KCl was added to the media, and the pH was adjusted to 5.8 using NH_4_OH, before autoclaving. A mixture of auxotrophic supplements was added after autoclaving. For Rb^+^ uptake, intracellular K^+^ content and membrane potential measurements, yeast cells were grown overnight in a YNB, supplemented with 100 mM KCl to OD_600_ 0.3–0.4, then harvested and used for measurements, or additionally incubated for various times in a YNB-F supplemented with the indicated concentrations of KCl.

### 2.2. Plasmids

The plasmids used in this study are listed in Table S2 and the oligonucleotides used for their construction and verification are listed in Table S3. To construct centromeric plasmids, the *CEN6ARS4* fragment from pRS316 was amplified by PCR, using the oligonucleotides 2µ-CEN6ARS4_for and 2µ-CEN6ARS4_rev. The resulting DNA fragment was used to replace the 2µ sequence in the linearized YEp352- or pGRU1-derived plasmids via homologous recombination. Proper insertion was confirmed by diagnostic PCR. The plasmids that expressed Trk1 with amino-acid substitutions were constructed by site-directed mutagenesis (QuikChange II Site-Directed Mutagenesis Kit, Agilent Technologies, Santa Clara, CA, USA), using pCScTRK1 or pCScTRK1-GFP as templates. All substitutions were confirmed by sequencing.

### 2.3. Growth Assay

The growth of yeast cells was tested on solid media supplemented with 2% agar. The cells were pre-grown on YNB plates, supplemented with 100 mM KCl, and resuspended in sterile water to OD_600_ 0.6. Serial 10-fold dilutions were prepared and 3 µL aliquots were spotted on YNB-F plates, supplemented with the indicated concentrations of alkali-metal-cation salts, and incubated at 30 °C. The representative results of at least three independent repetitions are shown.

### 2.4. Estimation of Kinetic Parameters for Rb^+^ Uptake

The yeast cells were grown and incubated as described above, collected, washed with sterile water and resuspended in 50 mL of MES buffer (20 mM MES, 2% glucose, 0.1 mM MgCl_2_; pH adjusted to 5.8 with Ca (OH)_2_) to OD_600_ ~0.25. To measure the Rb^+^ uptake, RbCl was added to the cell suspension at time 0, and 5-mL samples were collected at 1 min intervals over 5 min. The samples were filtered through Millipore filters (0.8 µm pore size) and washed with 20 mM MgCl_2_. The cells on filters were subsequently extracted overnight in an extraction buffer (0.2 M HCl, 10 mM MgCl_2_, 0.2% KCl). The extracts were analyzed by atomic absorption spectrometry, and the initial rate of Rb^+^ uptake in nmoles per mg of dry weight of cells per minute was calculated [24]. Six different RbCl concentrations, ranging from 50 µM to 5 mM, were used for the potassium-starved cells, and six concentrations, ranging from 1 mM to 20 mM, for non-starved cells. For the estimation of the K_T_ and V_max_ kinetic parameters, the values corresponding to the initial rate of Rb^+^ uptake obtained for individual external Rb^+^ concentrations were plotted on a Lineweaver–Burk plot. For rough estimates of the kinetic parameters, only the 50 µM and 5 mM concentrations were used.

### 2.5. Measurements of K^+^ Content

The yeast cells were grown and incubated as described above, collected, washed with sterile water and resuspended in an MES buffer (20 mM MES, 2% glucose, 0.1 mM MgCl_2_; pH adjusted to 5.8 with Ca(OH)_2_) to OD_600_ ~0.4. Three 1-mL samples were immediately withdrawn, filtered through Millipore filters (0.8 µm pore size), washed with 20 mM MgCl_2_ and extracted overnight in a K^+^-free extraction buffer (0.2 M HCl, 10 mM MgCl_2_). The extracts were analyzed by atomic absorption spectrometry and the total K^+^ content in nmoles per mg of dry weight of cells was calculated, as previously described [25].

### 2.6. Estimation of Relative Membrane Potential

A fluorescence assay, based on the potential-dependent redistribution of the probe diS-C3(3) (3,3′-dipropylthiacarbocyanine iodide), was used for the estimation of the relative membrane potential, as previously described [26]. Cells were grown and incubated as described above, washed with sterile water and resuspended in an MES buffer (20 mM MES, 0.1 mM MgCl_2_; pH adjusted to 5.8 with Ca(OH)_2_) to OD_600_ 0.2. The probe was added to a 2 × 10^−8^ M final concentration from a 10^−4^ M stock solution in ethanol. The fluorescence emission spectra (λ_ex_ = 531 nm and λ_em_ = 560–590 nm) of the cell suspensions were measured in a Fluoromax 4 spectrofluorometer equipped with a Xenon lamp. The staining curves are presented as the time-dependent spectral shift of the wavelength maximum (λ_max_) of diS-C3(3) fluorescence emission. Three independent measurements were performed for each strain and condition; either representative staining curves or mean values of λ_max_ reached after 50 min of staining are shown.

### 2.7. Fluorescence Microscopy

The yeast expressing GFP-tagged Trk1 versions were used for visualizing the subcellular localization of Trk1. Cells were either grown in a YNB supplemented with 100 mM KCl or in a YNB-F supplemented with various KCl concentrations to OD_600_ ~1, and visualized using an Olympus Bx53 microscope (Olympus, Hamburg, Germany) and captured with an Olympus DP73 camera (Olympus, Hamburg, Germany). The excitation of 460 nm and emission of 515 nm was used to visualize the GFP-tagged Trk1 proteins. Differential interference contrast (DIC) was used for the visualization of whole yeast cells.

### 2.8. Bioinformatics

Sequence alignment was performed using the multiple sequence alignment tool MUSCLE (EMBL-EBI, HINXTON, UK) with the default parameters. For the visualization of the positions of the substituted amino acids, the 3D atomic-scale model of *S. cerevisiae* Trk1 from reference [11] was used. The visualization was obtained using USFC ChimeraX version 1.1 (University of California, San Francisco, CA, USA) [27].

### 2.9. Statistics

All data were analyzed in Microsoft Excel 2010 (Microsoft, Prague, Czech Republic) or GraphPad Prism version 9.1.0. (GraphPad Software, San Diego, CA, USA), and *p*-values were calculated using the two-tailed Student’s T-test. All experiments were performed at least three times and the mean values with standard deviation are shown.

## 3. Results

### 3.1. Role of Extracellular and Intracellular K^+^ Content in Changes in Trk1 Affinity

**Figure 1:**
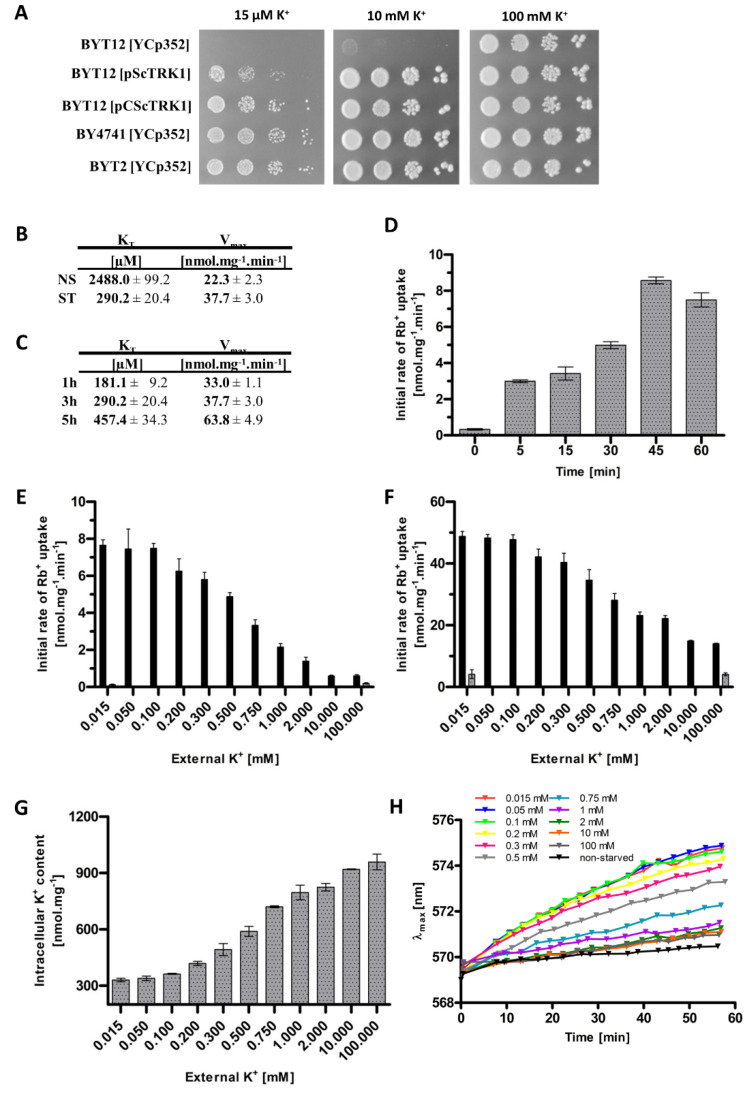
Changes in Trk1’s transport capacity, intracellular K^+^ content and membrane potential upon shifts in external K^+^ concentration. (**A**) Growth of strains BYT12 (trk1Δ trk2Δ), BY4741 wt and BYT2 (trk2Δ), transformed either with the YCp352 empty vector, pScTRK1 multicopy vector or pCScTRK1 centromeric vector, on YNB-F plates supplemented with indicated concentrations of K^+^. Pictures were captured after 3 days of incubation at 30 °C. (**B**) Kinetic parameters of rubidium uptake in potassium non-starved (NS) and starved (ST) cells. BYT12[pCScTRK1] cells were grown as described in Section 2. ST-cells were further incubated in a YNB-F (15 μM K^+^, conditions of potassium starvation) for an additional 3 h. Rb^+^ uptake was measured and kinetic parameters calculated as described in Section 2. (**C**) Time course of the changes in kinetic parameters of rubidium uptake. BYT12[pCScTRK1] cells were grown as in (**B**) and starved of potassium in YNB-F for 1, 3 and 5 h. The estimation of kinetic parameters was the same as in (**B**). (**D**) Initial rates of 50 µM Rb^+^ uptake in BYT12[pCScTRK1] cells within the first hour of K^+^ starvation. Cells were grown overnight as in (**B**), transferred to a YNB-F, and the uptake of Rb^+^ was measured immediately and after 5, 15, 30, 45 and 60 min. (**E**) Initial rates of 50 µM Rb^+^ uptake in BYT12[pCScTRK1] (black columns) and BYT12[YCp352] (grey columns) cells incubated in media with various K^+^ concentrations for 1 h. Cells were grown as in (**B**) and then incubated in a YNB-F supplemented with the indicated concentrations of K^+^. (**F**) Initial rates of 5 mM Rb^+^ uptake in BYT12 [pCScTRK1] (black columns) and BYT12 [YCp352] (grey columns). The experiment was performed as in (**E**). (**G**) Intracellular K^+^ content in BYT12[pCScTRK1] cells after incubation for 1 h in YNB-F media with various K^+^ concentrations. Cells were incubated as in (**E**), 3 aliquots of cell suspension were withdrawn immediately, and the concentration of K^+^ was estimated as described in Section 2. (**H**) Relative membrane potential of BYT12[pCScTRK1] cells after incubation for 1 h in YNB-F media with various K^+^ concentrations. Cells were grown overnight and incubated as in (**E**), then resuspended in an MES buffer, diS-C3(3) probe was added and the fluorescence emission spectra were measured. Staining curves are presented as the time-dependent spectral shift of the wavelength maximum (λ_max_) of diS-C3(3) fluorescence emission. Non-starved cells (black curve) are cells probed without 1-h incubation in a YNB-F.

To select the appropriate strain for the characterization of Trk1’s kinetic parameters, we first compared the growth of five strains. The wild-type BY4741, which possesses chromosomally encoded *TRK1* and *TRK2* genes, and the BYT12 strain, which lacks both these genes, were transformed with the empty vector YCp352. The other three strains lacked the *TRK2* gene and expressed *TRK1,* either from its original chromosomal locus (BYT2 with the empty plasmid YCp352) or from centromeric (BYT12[pCScTRK1]) or multicopy (BYT12[pScTRK1]) plasmids, respectively. As shown in Figure 1A, the lack of both *TRK* genes rendered the cells unable to grow in media with low KCl concentrations. In addition, surprisingly, the expression of Trk1 from a multicopy plasmid (BYT12[pScTRK1]) resulted in significantly worse growth on limiting K^+^ concentrations, compared to the other strains possessing *TRK1* genes. It is worth noting that most of the published results dealing with the characterization of Trk1 properties have so far been obtained with cells expressing Trk1 from various multicopy plasmids. As chromosomal *TRK1* is thought to be expressed at a low and constitutive level, we decided to use the BYT12 strain (lacking chromosomal copies of both *TRK* genes), transformed with a centromeric plasmid containing the *TRK1* gene behind a well-characterized, weak and constitutive *NHA1* promoter. We believed that this approach would prevent possible changes in *TRK1* expression in a varying potassium environment and, thus, changes in Trk1 transport capacity.

Next, we confirmed that we could detect the dual affinity of Trk1, i.e., a much higher affinity in potassium-starved cells than in cells growing at high potassium concentrations (a YNB supplemented with 100 mM KCl). To estimate the kinetics of transport via Trk1, we employed the usually used potassium analogue rubidium ([8], cf. Materials and Methods). Figure 1B summarizes the obtained results and shows that starving cells for potassium in the YNB-F (with only 15 µM KCl) for 3 h increased the affinity of Trk1 almost 10-fold, compared to non-starved cells (K_T_ ~290 and 2500 µM, respectively), while the maximum velocity increased from 23.3 to 37.7 nmoles.mg^−1^ min^−1^.

To elucidate how the affinity changes with the length of potassium starvation, we estimated the kinetic parameters after 1 and 5 h of starvation. We found that the highest affinity was observed after only 1 h, and with prolonged incubation at low potassium conditions, it surprisingly slightly decreased, while the V_max_ increased over time, being about twice as large after 5 h as after 1 h (Figure 1C). To observe in more detail how quick the affinity change is, we measured the initial uptake of 50 µM Rb^+^ immediately after transferring the cells to a starvation medium, and also after 5, 15, 30, 45 and 60 min. As shown in Figure 1D, the uptake of Rb^+^ was clearly measurable after only 5 min, which suggested that the lack of potassium is quickly sensed by the cell, and the affinity of Trk1 rapidly adapts to the new potassium conditions. The majority of previous studies that focused on the Trk1 affinity switch were performed after 3 h of potassium starvation; however, taking into account the results we obtained (Figure 1C,D), we decided to starve the cells for only 1 h in most of the following experiments.

Our first experiments confirmed the role of external potassium in the regulation of the kinetic parameters of Trk1. Whether directly or indirectly, the low concentration of external potassium upregulates Trk1’s capacity to import potassium/rubidium. However, the exact dynamics of the dependence of the Trk1 transport capacity on the changes in the amount of external potassium is unclear. Considering the ‘dual affinity’ model, ergo, switching between low and high-affinity states, we assumed the existence of a threshold amount of external potassium above (or below), which the Trk1 protein would switch to one of the two states. In order to identify this threshold concentration, we measured the initial rates of either 50 µM or 5 mM Rb^+^ uptake after 1 h of incubation in a YNB-F supplemented with 11 different concentrations of potassium, up to 100 mM. The obtained results are summarized in Figure 1E,F, respectively. Interestingly, we did not observe a sharp increase/decrease in the initial rates of Rb^+^ uptake upon incubation, in media supplemented with a specific concentration of potassium, i.e., a threshold concentration for the affinity switch, which we had expected according to the ‘dual affinity’ model. We instead detected a gradual change in the initial uptake rate depending on the availability of external potassium. The same course of gradual change of uptake was observed for both the low (50 µM) and high (5 mM) concentrations of the substrate (Figure 1E,F). When the rough estimates of the kinetic parameters were calculated (Table 1), it was evident that both affinity and maximum velocity increased gradually, with the decrease in external potassium concentration. Trk1 exhibited its maximum capacity to import Rb^+^ after the incubation of cells in media supplemented with ≤0.1 mM KCl, and minimal capacity after incubation in media supplemented with ≥10 mM KCl. As a negative control, we used the same cells transformed with an empty vector, and the obtained results showed that the significant uptake of Rb^+^ was only observed in cells expressing Trk1 (Figure 1E,F).

Next, we wanted to estimate whether and how much the changes in the external potassium concentration affect the intracellular potassium content, and whether the changes in the intracellular potassium are reflected in the changes in the kinetic parameters. These experiments were based on the observation that the depletion of external potassium leads to a gradual loss of intracellular potassium content [28]. Figure 1G shows that the incubation of cells (pre-grown in the presence of 100 mM KCl) in a YNB-F supplemented with various external concentrations of potassium, for 1 hour, dramatically changed their intracellular potassium content; the lower the external concentration, the deeper the observed decrease in intracellular potassium content. By extrapolating the associations between the decrease in external potassium concentration and increase in Trk1 affinity on one hand, and the dependency of intracellular K^+^ content on the extracellular potassium conditions on the other, we were able to highlight a possible relation between the intracellular potassium concentration and the regulation of Trk1 affinity. The external K^+^ concentrations, at which we observed approximate maximal and minimal concentrations of intracellular potassium, closely correlated with the external K^+^ concentrations, at which we detected the lowest and highest initial rates of Rb^+^ uptake, respectively. After incubation in the presence of ≥10 mM KCl, we observed the maximum intracellular potassium content and minimal initial rate of Rb^+^ uptake. Vice versa, after incubation in ≤0.1 mM KCl, we detected the lowest intracellular potassium content and measured the maximal initial rates of Rb^+^ uptake (Figure 1E–G).

As Trk1 contributes to the maintenance of appropriate potential across the plasma membrane, changes in K^+^ transport across the plasma membrane, and consequently potassium content, should be reflected in the changes in cell membrane potential. To confirm this presumption, we estimated the differences in relative membrane potential of cells pre-grown with 100 mM KCl and then incubated in a YNB-F supplemented with various KCl concentrations for 1 hour, similarly for the experiments summarized in Figure 1E–G. We monitored the changes in relative membrane potential with a potentiometric fluorescence probe and the obtained results (Figure 1H) confirmed our presumption. The incubation of cells in the presence of low KCl concentrations (15–100 µM) led to the highest relative hyperpolarization, whereas increasing the external KCl concentrations (above 100 µM) resulted in a more and more pronounced decrease in the relative membrane potential. We also observed a small difference in the relative membrane potential between the cells pre-grown in 100 mM KCl and probed directly, and the cells pre-grown in 100 mM KCl and transferred to a fresh YNB-F supplemented with 100 mM KCl for 1 h (black and dark grey curves in Figure 1H, respectively). This difference may serve as a positive control of our method, as it has been repeatedly shown that the transfer of cells to fresh media (with a higher concentration of glucose) leads to an immediate activation of Pma1, measurable as both an alkalinization of the cytosol and as an increase in the relative membrane potential (e.g., references [29,30]). In summary, the use of a potentiometric probe confirmed our assumption that a change in the external/internal concentration of potassium, reflected in the changes in Trk1 affinity, correlates with the changes in the membrane potential.

According to all the results summarized in Table 1, Trk1 does not, in fact, switch between low- and high-affinity states, but rather the kinetic parameters slide along with the changes in the external and internal potassium concentrations and follow the changes in membrane potential, suggesting the possibility of up to three signals being involved in the regulation of Trk1’s transport capacity.

**Figure 2:**
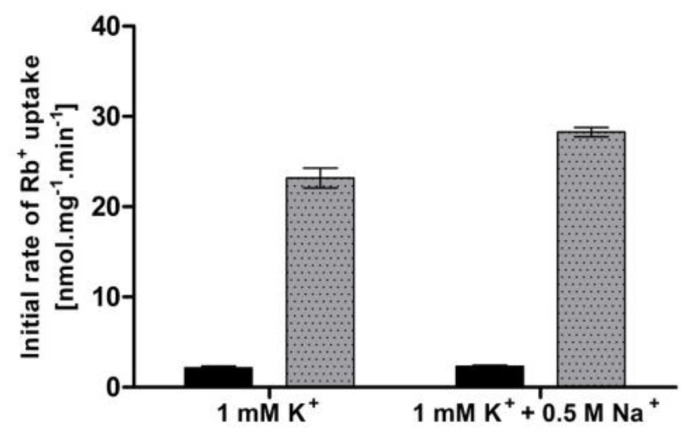
Effect of high external Na^+^ concentration on Trk1-mediated Rb^+^ uptake. BYT12 [pCScTRK1] cells were grown overnight as described in Section 2, and then incubated in a YNB-F supplemented with either 1 mM K^+^ or 1 mM K^+^ and 0.5 M Na^+^ for 1 h. Initial rates of 50 µM (black columns) and 5 mM (grey columns) Rb^+^ uptake were measured, as described in Section 2.

Apart from the limitations in external potassium concentration, an elevated external concentration of sodium has also been suggested to provoke a high-affinity state of Trk1 [16]. To verify whether we could observe a change in Trk1 affinity upon the addition of salt, we measured the uptake of Rb^+^ (50 µM or 5 mM, respectively) in the presence/absence of NaCl. The cells were pre-grown with 100 mM KCl and incubated in a YNB-F supplemented with 1 mM KCl (to ensure a medium-affinity state of Trk1 in conditions w/o NaCl) and with/without 0.5 M NaCl for 1 hour. In contrast to previously published data [16], we detected no significant difference in the initial rates of 50 µM Rb^+^ uptake, and only a very small difference in the 5 mM Rb^+^ uptake between cells incubated with and without NaCl (Figure 2). When we used the measured initial rates to calculate the rough estimates of K_T_, the obtained values were very similar (approx. 550 µM after incubation in 1 mM potassium and 630 µM after incubation in 1 mM potassium + 0.5 M sodium). Our results did not confirm the increase in Trk1 affinity because of the exposure to a high concentration of toxic sodium in cells with the BY4741 genetic background.

### 3.2. Trk1 Affinity Is Directly Proportional to the Level of Plasma-Membrane Potential

To characterize in more detail the regulatory signal for the aforementioned changes in Trk1 capacity to import potassium, we decided to compare the kinetic parameters, intracellular potassium content and relative membrane potential of four strains, expressing *TRK1* under different conditions. These were BY4741[YCp352], BYT2[YCp352] with chromosomal copies of *TRK1*, and two BYT12 strains lacking chromosomal copies of both *TRK* genes and transformed with a centromeric (pCScTRK1) or multicopy (pScTRK1) plasmids, respectively. Although these strains exhibit differences in the ability to grow on low potassium (Figure 1A) and in some physiological parameters [25], we presumed that the fundamental mechanism of Trk1 regulation should be similar; therefore, by extracting a common pattern of the correlation of the changes in the Trk1 kinetic parameters and changes in either the intracellular potassium content or relative membrane potential, the dominant regulatory force could be identified.

**Figure 3:**
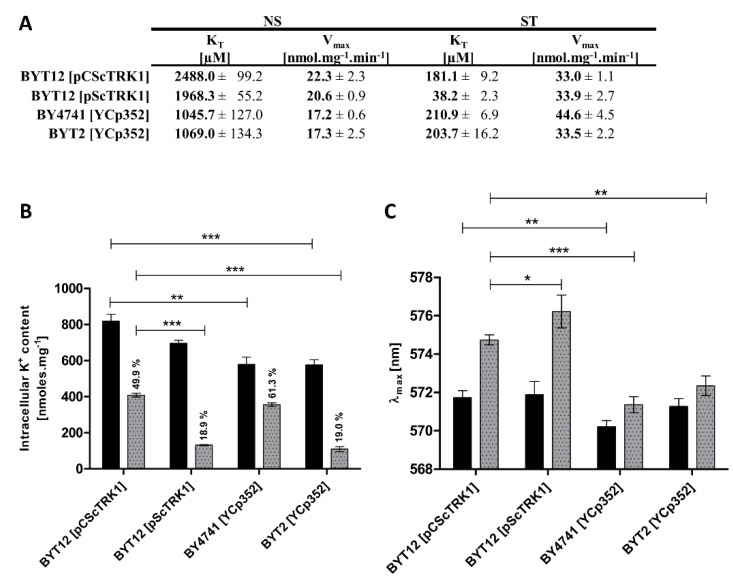
Differences in kinetic parameters, intracellular K^+^ content and membrane potential among strains. Cells expressing *TRK1* either from chromosome (BY4741[YCp352] and BYT2 (*trk2*Δ)[YCp352]) or from centromeric (BYT12[pCScTRK1]) and multicopy plasmids (BYT12[pScTRK1]), respectively, were grown as described in Section 2, and used directly (non-starved (NS) cells) or first incubated in a YNB-F (15 μM K^+^, conditions of potassium starvation) for 1 h (starved (ST) cells). (**A**) Differences in kinetic parameters of Rb^+^ uptake. Initial uptake rates and kinetic parameters were estimated as in Figure 1B. (**B**) Intracellular K^+^ content. Three aliquots of NS (black columns) or ST (grey columns) cell suspension were withdrawn and the concentration of K^+^ in cells was estimated as described in Section 2. (**C**) Relative membrane potential. The membrane-potential measurement was performed as in Figure 1G with NS (black columns) and ST (grey columns) cells. The λ_max_ reached after 50 min of staining is shown for each strain. Significant differences in (**B**,**C**) are indicated with asterisks (* *p* < 0.05; ** *p* < 0.01; *** *p* < 0.001).

The comparison of kinetic parameters yielded an unexpected degree of variety in Trk1’s affinity in both potassium non-starved cells and starved cells (Figure 3A). In non-starved cells (NS), the strains expressing *TRK1* from the chromosome (BY4741 and BYT2) exhibited a significantly higher affinity (K_T_ values ~1000 µM) than the strains expressing *TRK1* from plasmids (K_T_ around 2000 µM for the multicopy and around 2500 µM for the centromeric plasmids, respectively). The maximum velocity of rubidium uptake was similar in all four strains (~17–22 nmol.mg^−1^ min^−1^). In cells starved of potassium for 1 h (ST), we observed a similar K_T_ for cells expressing *TRK1* from the centromeric plasmid or chromosome (approx. 200 µM), and a strikingly high affinity (38 µM) of Trk1 in cells transformed with the multicopy pScTRK1 plasmid. In addition, we detected comparable V_max_ values, which were also surprising, as the V_max_ is predominantly determined by the total number of active transporters in the membrane. According to this principle, we would expect the V_max_ to be highest in the strain BYT12[pScTRK1]. We only observed an increase in the V_max_ of BY4741 cells, which could be attributed to a more pronounced contribution of the Trk2 system under starvation conditions. The discrepancy between the observed changes in affinity and changes in maximum velocity suggested that these two parameters are regulated differently. In summary, our results clearly showed a difference in the intensity of the response to potassium starvation, mediated by an increase in the affinity of Trk1. While both strains expressing Trk1 from a chromosome exhibited a comparable increase in affinity upon potassium starvation (5.0-fold for BY4741 and 5.2-fold for BYT2), in the BYT12[pCScTRK1] strain the Trk1’s affinity increased 13.7-fold, and Trk1 in the BYT12[pScTRK1] strain exhibited a 51.5-fold increased affinity for Rb^+^ (Figure 3A). The above-mentioned differences also contradicted the direct role of external potassium in the regulation of the affinity of Trk1. For all four strains, the external concentration of potassium was identical throughout the experiments (both overnight cultivation and the subsequent starvation period), but the values of K_T_ before and after potassium starvation were vastly different.

To distinguish whether the dominant role in the affinity level is played by the intracellular potassium content or membrane potential, we tested the correlation between the degree of changes in affinity and the degree of changes in intracellular potassium content and membrane potential in the four strains, after 1 h of potassium starvation. As shown in Figure 3B, the intracellular potassium content already exhibited some differences in non-starved cells, i.e., cells grown in the presence of 100 mM KCl (conditions under which the activity of Trk1 is dispensable; cf. Figure 1A, strain BYT12[YCp352]). The cells expressing Trk1 from plasmids (BYT12 [pCScTRK1] or [pScTRK1]) accumulated a higher amount of potassium (820 and 700 nmol/mg, respectively), compared to the 580 nmol/mg in strains BY4741 and BYT2 with chromosomal copies of *TRK1.*

Considering the possibility of the internal potassium content being the main determinant of the affinity of Trk1, the observed differences between the internal potassium content were in line with the differences in the affinity of non-starved cells (Figure 3A). Thus, the higher affinity detected in BY4741 and BYT2 strains could be a consequence of the accumulation of a lower amount of potassium in these cells. Nevertheless, the results obtained after one hour of potassium starvation disproved the general validity of this conclusion. Upon starvation, these were the cells expressing *TRK1,* either from a multicopy plasmid or cells with chromosomally encoded *TRK1* and lacking the *TRK2* gene, which similarly lost more than 80% of their intracellular potassium content (Figure 3B), but whose affinity did not increase in a similar way (Figure 3A). This result contradicted the presumed major role of internal potassium in the regulation of the affinity of Trk1, but simultaneously revealed a novel observation that Trk2 contributes significantly to potassium acquisition in starving cells, plays a role in the regulation of intracellular potassium content (cf. V_max_ and K^+^ content of BY4741 and BYT2 cells in Figure 3A,B, respectively), and its absence leads to a relative hyperpolarization of both non-starved and starved cells (Figure 3C).

Furthermore, we measured the changes in relative membrane potential in non-starved and starved cells. As shown in Figure 3C, the changes in λ_max_ (as an indicator of the level of the relative values of plasma-membrane potential) fully corresponded to the K_T_ changes during potassium starvation. Table 2 summarizes all the obtained results and shows a good correlation between the level of increase in membrane potential and the level of increase in Trk1’s affinity.

In summary, the increase in membrane potential upon potassium starvation seems to play a dominant role in the regulation of changes in Trk1’s affinity, and not the external or internal potassium content.

### 3.3. Role of Short P-Helices in Trk1 Activity and Affinity Adjustment

In the experiments described above, we focused on finding the potential regulator of Trk1’s affinity at the cellular level. To further elucidate how the changes in affinity are interconnected with protein structure, we focused on characterizing the role of one of the Trk1 amino-acid residues (Leu949) in the affinity changes.

**Figure 4:**
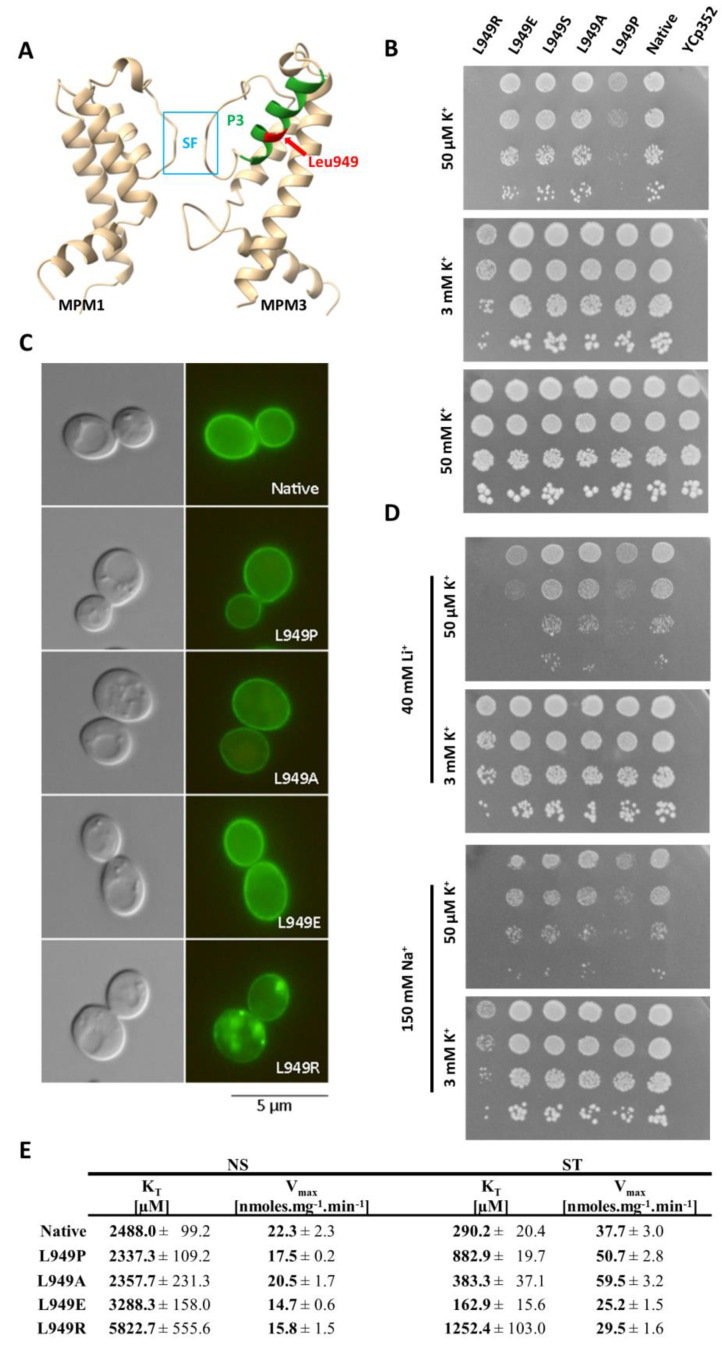
Effects of Leu 949 substitution. (**A**) Localization of Leu949 within a 3D model of Trk1 protein. P-helix 3 is shown in green, the position of Leu949 in P3 is highlighted in red, and the part of the selectivity filter (SF) in blue. For better clarity, only MPM domains 1 and 3 are shown. (**B**) Growth of BYT12 cells without *TRK1* (BYT12[YCp352]) or with centromeric vector harbouring *TRK1* (native) and its mutated versions on YNB-F plates supplemented with K^+^, as indicated. Images were captured after 5 days of incubation at 30 °C. (**C**) Localization of GFP-tagged Trk1 and its mutated versions in BYT12 cells. Cells were grown and viewed as described in Section 2. (**D**) Growth of cells on YNB-F plates supplemented with indicated concentrations of K^+^, Na^+^ and Li^+^. The order of strains and length of incubation were the same as in (**B**). (**E**) Kinetic parameters of native Trk1 and its mutated versions after 3 h of K^+^ starvation. Cells were grown as described in Section 2. ST cells were incubated in a YNB-F for an additional 3 h. Rb^+^ uptake was measured and kinetic parameters were estimated as described in Materials and Methods.

According to a 3D model of *S. cerevisiae* Trk1, Leu949 is localized approximately in the middle of a short P-helix (P3) of the third MPM motif, and is, thus, situated close to the region of the selective filter (SF) and potassium binding site (Figure 4A and reference [11]). We, therefore, assumed that the substitutions of Leu949 could have a significant effect on the conformation of helix P3 and consequently on the structure of the entire region of the selectivity filter, which could presumably affect potassium uptake as such. To more closely characterize not only the mutation L949P, but also the importance of leucine at this position, we performed site-directed mutagenesis replacing Leu949 with proline, alanine, glutamic acid, arginine, and serine. The BYT12 cells were transformed with the resulting plasmids (cf. Table S2), and the effects of these substitutions on the functionality, subcellular localization and kinetic parameters of Trk1 were studied.

The tests on the plates supplemented with various concentrations of potassium (Figure 4B) revealed that the growth of cells expressing the L949P version of Trk1 was significantly diminished at low potassium concentrations (50 µM; native Trk1 in high-affinity state), but not on plates supplemented with 3 mM KCl (native Trk1 with the affinity close to low-affinity state). This result was in line with the assumption that the substitution L949P specifically affected the increase in Trk1’s affinity upon potassium limitations. We detected no or minimal changes in growth on 50 µM KCl for substitutions L949A, L949S and L949E, which led to the conclusion that the effect of L949P substitution is, in fact, caused by the introduction of proline, rather than the absence of leucine at position 949. Finally, the introduction of a positively charged Arg residue into position 949 led to a complete inhibition of growth on low potassium and significantly diminished growth on the plates supplemented with 3 mM KCl (Figure 4B).

As shown in Figure 4C, native Trk1-GFP together with the mutated versions L949P, L949A and L949E were localized primarily in the plasma membrane;therefore, the effects of these mutations could be attributed to the altered functionality of Trk1, rather than to the changes in subcellular localization. On the other hand, the introduction of positively charged arginine into position 949 led to a substantial alteration in Trk1’s subcellular localization (Figure 4C). This effect was most likely due to defects in the folding of Trk1 and the subsequent partial retention within the secretory pathway, which in turn resulted in diminished Trk1 activity observed on a whole-cell level.

Some members of the SKT family of potassium transporters were shown to also transport small monovalent cations [31]. Thus, we further tested whether the introduced mutations could affect the size of the binding site and thereby change Trk1’s strict substrate specificity, e.g., allowing the binding and transport of smaller Na^+^ and Li^+^ cations. Even though we detected a slight overall negative effect of the high external concentrations of Na^+^ and Li^+^ in the presence of low concentrations of potassium (50 µM) on the growth of all the tested strains (Figure 4D), the growth pattern was, in fact, similar to the pattern observed for the strains grown only in the presence of 50 µM KCl (Figure 4B,D). The apparent exceptions were cells expressing the Trk1 mutant L949E. The introduction of a negative charge led to an increased sensitivity to Li^+^ in the presence of low KCl (but not with 3 mM KCl), suggesting that when there is a sufficiently high ratio of external concentrations of Li^+^ and K^+^, the L949E version could possess an increased capacity to transport lithium, but not sodium (Figure 4D).

Finally, to complete the characterization of the Leu949 mutants and to confirm the effect of L949P substitution on Trk1 affinity, we estimated the kinetic parameters of the non-starved and starved cells (summarized in Figure 4E). The obtained results confirmed the inability of the L949P mutant to properly increase its affinity, since this version of Trk1 exhibited approximately three-fold higher K_T_ than the native form after starvation. Interestingly, in non-starved cells, the K_T_ values for the native and L949P versions were similar, showing that the substitution L949P did not disrupt the functionality of Trk1 as such, but rather specifically the ability to shift to a higher affinity upon potassium starvation. The kinetic parameters of the L949A mutant confirmed the negligible effect of this mutation observed in the drop tests. These results attributed all the negative effects of the L949P substitution to the introduction of a proline, rather than to the loss of a leucine in the 949 position. The results obtained for the L949R version also fully corresponded to the results of the drop tests. We detected a substantial decrease in the affinity of the mutated Trk1 in both non-starved and starved cells, suggesting that the introduction of a positive charge into position 949 has a generally negative effect on Trk1 activity (Figure 4E). We observed surprising effects on Trk1 affinity with the mutation L949E. Whereas the affinity of this mutant was significantly lower than that of the native Trk1 in non-starved cells (K_T_ approx. 3300 vs. 2500 µM, respectively; Figure 4E), we detected the opposite effect of this mutation in starved cells, in which the affinity was approx. 160 µM, i.e., the highest of all experiments performed with Trk1 expressed from a centromeric plasmid (compare Figure 1B,C, Figure 2B and Figure 4E).

Our results confirmed that the introduction of proline in the P3-helix at position 949 negatively affects the proper increase in affinity as a response to potassium starvation, and showed that the introduction of charged amino acids in this position also changes the transport properties of Trk1.

**Figure 5:**
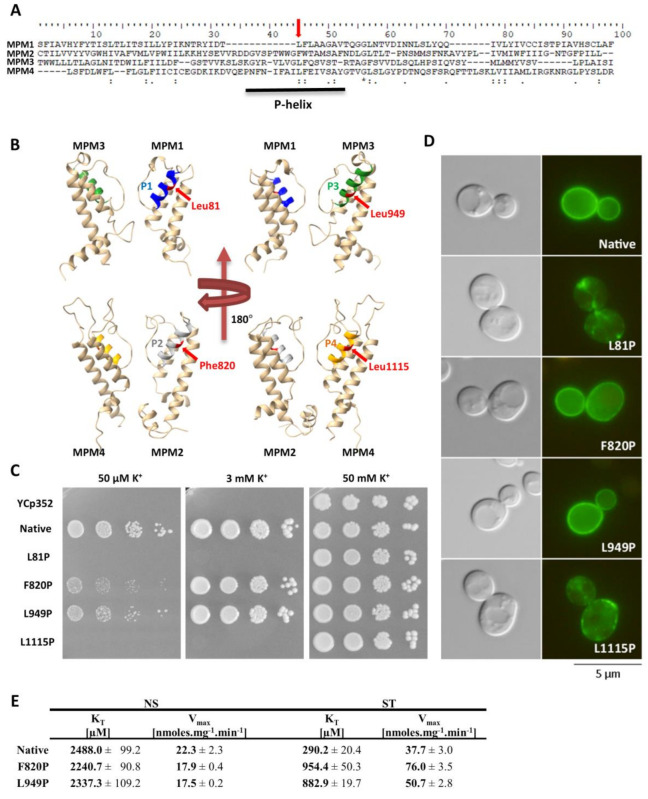
Effects of introduction of proline into P-helices. (**A**) Sequence alignment of Trk1’s MPM domains 1–4. Leu949 and corresponding amino acids in MPM 1, 2 and 4 are indicated with red arrows. P-helices within MPM domains are underlined. (**B**) Positions of amino acids Leu81, Phe820, Leu949 and Leu1115 within 3D model of Trk1 protein. P-helix 1 is highlighted in blue, 2 in grey, 3 in green and 4 in orange. Studied amino-acid residues are shown in red. For better clarity, only two opposing MPM domains are shown. (**C**) Growth of BYT12 cells without *TRK1* (BYT12 [YCp352]) or with centromeric vector harbouring *TRK1* (native) and its mutated versions on YNB-F plates supplemented with K^+^ as indicated. Images were captured after 5 days of incubation at 30 °C. (**D**) Localisation of GFP-tagged Trk1 and its mutated versions in BYT12 cells. Cells were grown and viewed as described in Section 2. (**E**) Kinetic parameters of native Trk1 and its mutated versions after 3 h of K^+^ starvation. Both non-starved (NS) and starved (ST) cells were grown as described in Section 2. ST cells were incubated in a YNB-F for an additional 3 h. Rb^+^ uptake was measured and kinetic parameters were estimated as described in Section 2.

As Trk1 contains four MPM domains, we decided to test the effects of similar mutations (the introduction of a prolyl residue) in the other three P-helices. The amino-acid residues for substitution were selected according to a sequence alignment of all four MPM domains (Figure 5A). The amino-acid residues corresponding to Leu949 were Leu81, Phe820 and Leu1115 (Figure 5B), and their replacement with proline was performed by site-directed mutagenesis of the *TRK1* gene in the pCScTRK1 and pCScTRK1-GFP plasmids, respectively (Table S2).

By testing the growth of the cells expressing mutated *TRK1* versions on plates supplemented with various concentrations of potassium, and by observing the subcellular localisation of GFP-tagged Trk proteins, we detected two distinct effects of proline introduction into P-helices. The introduction into P-helices number 1 and 4 (L81P and L1115P) led to a complete loss of cell ability to grow at low potassium concentrations (50 µM and 3 mM; Figure 5C). This growth inhibition was most likely due to an almost complete mislocalisation of these mutated versions of Trk1 (Figure 5D).

For the substitution F820P (MPM2), we observed effects similar to L949P. The cells expressing the F820P version were relatively sensitive to low potassium concentrations (Figure 5C), but the subcellular localization of this mutated Trk1 version remained unaltered (Figure 5D). The kinetic parameters of the F820P version in non-starved cells were comparable to those of the native and L949P versions, respectively (Figure 5E). For potassium-starved cells expressing Trk1 F820P, we observed a slightly higher inability to reach the maximum affinity than for the L949 version (Figure 5E). As the growth of cells expressing this version on low potassium was also slightly more inhibited than the growth of cells with the L949P version (Figure 5C), we concluded that the introduction of proline into P-helix 2 has an even more significant effect on Trk1’s ability to increase its affinity, than the introduction of proline into P-helix 3.

**Figure 6:**
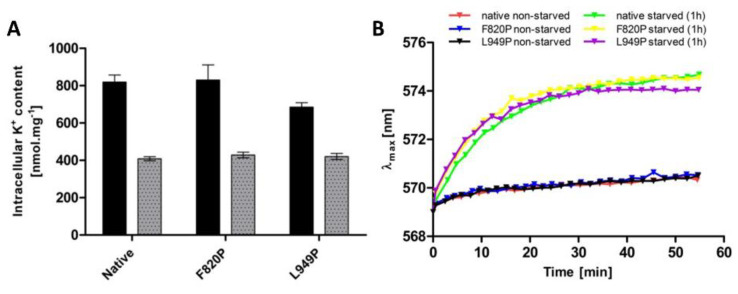
Intracellular K^+^ content and relative membrane potential in BYT12 cells with native, L949P and F820P Trk1 versions. Cells were grown as described in Materials and Methods, and used directly (non-starved cells), or first incubated in a YNB-F for 1h (starved cells). (**A**) Intracellular K^+^ content was estimated in non-starved (black columns) and starved (grey columns) cells as described in Section 2. (**B**) Relative membrane potential was estimated as described in Section 2.

As mentioned in previous sections, intracellular potassium content and membrane potential might play a role in the regulation of the changes in Trk1 kinetic parameters. To distinguish whether the mutations F820P and L949P prevent the increase in Trk1 affinity due to alterations in the transporter’s functionality, or whether they disrupt overall potassium homeostasis, thus, affecting the regulatory signal for an increase in affinity, we estimated both the intracellular potassium content and relative membrane potential in non-starved and starved cells expressing the native and mutated versions, respectively. As shown in Figure 6, both the intracellular potassium content and accumulation of the potentiometric probe were almost the same in cells expressing either the native or F820P or L949P versions of Trk1. These results led us to conclude that the inability of Trk1, with the substitutions F820P and L949P, to properly increase its affinity upon potassium starvation is a consequence of the alterations in protein structure and functionality, rather than the absence of a regulatory signal.

## 4. Discussion

The central role of Trk1 in yeast potassium uptake has been known for many years, and its ability to switch its affinity according to the available potassium has been demonstrated many times. Even though a considerable amount of data has been generated in previous studies, a comprehensive picture of the regulation and mechanism of the affinity changes remains unavailable.

There are a few examples of transporters with the ability to switch their affinity for substrates. The most thoroughly described is the plant nitrate importer Nrt1.1. Upon a sharp decrease in external nitrate concentration, Nrt1.1 switches to a high-affinity state, simultaneously decreasing its K_T_ and V_max_ [32]. It is the phosphorylation of one threonyl residue (Thr101) that leads to a dissociation of the Nrt1.1 dimers with elevated structural flexibility and increased affinity of monomers for nitrate uptake [33,34,35]. While the common pattern, i.e., an increase in affinity as a result of the limited availability of substrate, seems to be similar between Nrt1.1 and Trk1, there are a couple of notable differences. First, the most significant difference between Nrt1.1 and Trk1 are the dynamics of the K_T_ changes. Nrt1.1 switches between only two affinity states, which has also been originally presumed for Trk1 [13,15]. However, according to our results, Trk1 does not switch between only two states, but rather gradually adjusts its affinity according to the availability of external potassium (Figure 1E,F and Table 1).

Second, in contrast to Nrt1.1, the Trk1’s affinity change is accompanied by a significant increase in V_max_. We observed the V_max_ in the high-affinity state to be almost double that of the V_max_ in the low-affinity state (Figure 1B). In addition, the time course of the V_max_ increase differs from the time course of the increase in affinity (Figure 1C), suggesting that the two processes are, at least, partially independent. The relative independence of the observed changes in both kinetic parameters is in contrast to the documented cases of the affinity switch of other transporters, in which the increase in affinity is accompanied by a significant decrease in maximum velocity [33,36,37]. The explanation of this phenomenon is that the high-affinity state leads to a stronger bond between the binding site and substrate, which in turn leads to an overall decrease in transport velocity. We cannot exclude the possibility that a similar phenomenon exists for Trk1, and that the high-affinity-induced decrease in maximum velocity is masked by the elevation in the number of active transporters in the membrane, as a reaction to potassium starvation. There are a few potential mechanisms for an increase in the number of active transporters—increased gene expression, stimulation of the Trk1 molecule passage from later stages of the secretory pathway to the plasma membrane, or the activation of previously inactive plasma-membrane transporters. The stimulation of gene expression is an unlikely explanation, as *TRK1* was expressed from a constitutive promoter. Additionally, the expression of chromosomally-encoded *TRK1* is not responsive to changes in external potassium levels [2,38]. The stimulation of the secretion of transporters from the later stages of the secretory pathway [39] is again unlikely, as we did not observe a significant proportion of GFP-tagged Trk1 proteins to be retained in the secretory pathway in the high-potassium environment (Figure S2). Thus, the most likely explanation is the inactivity of a significant portion of proteins under high-potassium conditions and their gradual activation as a response to prolonged potassium starvation.

Taken together, our data demonstrate that the observed changes in whole-cell capacity to transport potassium, under conditions with a limited amount of this cation, consists of at least two partially independent events reflecting the availability of potassium, a gradual increase in Trk1’s substrate affinity, and a gradual elevation of the number of active transporters in the plasma membrane. This is in sharp contrast with the simple Nrt1.1 switching between a low-V_max_-low-K_T_ and a high-V_max_-high-K_T_ state. As a consequence, the excessive increase in the uptake of nitrate via a high-affinity Nrt1.1 is prevented by a substantial decrease in V_max_. A similar regulation for Trk1, i.e., existence in only two states (either high affinity and high velocity or low affinity and low velocity) would only be favourable in environments with either extremely high or extremely low concentrations of potassium. In environments with a potassium concentration close to the presumed threshold for the affinity switch, sudden and small shifts in the availability of potassium would lead to a substantial increase or decrease in potassium uptake, and inevitably, to momentous disturbances in potassium homeostasis. The precise adjustment of both kinetic parameters of Trk1 described here would render yeast cells viable over a broad range of external potassium concentrations, and enable them, to a certain extent, to compensate for rapid changes in environmental potassium, without significant disturbances to their internal potassium balance.

The external potassium concentration has been considered to be a pivotal signal in the regulation of Trk1’s affinity for a long time [13]. We confirmed this premise by observing a substantial decrease in K_T_ in cells starved of potassium, compared to non-starved cells (Figure 1B). The observed gradual increase in both the affinity and maximum velocity precisely copied the decrease in external potassium concentration (Figure 1E,F, Table 1). However, the assumption that Trk1 reacts exclusively and directly to the changes in the external potassium levels was contradicted by a comparison of the kinetic parameters and their changes in four strains cultivated and subsequently starved under the same conditions. The affinities and also the degree of changes in their Trk1 systems were vastly different (Figure 3A). We, therefore, conclude that, while the external potassium has an apparent effect on the uptake capacity of Trk1, this effect is indirect. Interestingly, in contrast to the values of K_T_, the values of V_max_ seemed to be mostly comparable among the four strains, supporting the hypothesis that the regulation of K_T_ and V_max_ upon potassium starvation is separate (Figure 3A).

Another potential signal initiating the transition of Trk1 to the high-affinity state could be a decrease in the internal potassium concentration [15]. Our results, which exhibit a connection between a gradual loss of potassium and an increase in both the affinity and maximum velocity, support this presumption (Figure 1G and Table 1). The potential mechanism of such regulation of Trk1 should involve protein(s) with intracellular potassium-binding domains (possibly Trk1 itself), which would bind intracellular potassium with a frequency proportional to its internal concentration. The binding of potassium would lead to conformational changes that result in activity alterations. Potassium-sensing proteins have been reported, e.g., a small cytosolic protein in *E. coli* [40]. However, this mechanism of Trk1’s affinity regulation is unlikely. Although potassium starvation leads to a loss of internal potassium in *S. cerevisiae*, presumably by export from the cytoplasm, the actual levels of potassium in the cytoplasm remain relatively stable, because they are constantly supplemented from potassium stocks in vacuoles [41]. Consequently, the intracellular domains of Trk1 are probably exposed to relatively constant amounts of cytosolic potassium; thus, the direct regulation of Trk1’s affinity by the amount of potassium in the cytosol seems unlikely. In addition, more data that contradict the regulatory role of internal potassium came from the comparison of the four strains. Even though we detected a comparable loss of internal potassium in strains BYT12[pScTRK1] and BYT2 [YCp352] upon potassium starvation (approx. 80%, Figure 3B), the values of K_T_ and their change also differed significantly (Figure 3A). Taken altogether, in addition to external potassium, our results also contradicted a direct role of internal potassium in the regulation of Trk1 affinity.

Finally, our data suggested another physiological parameter, membrane potential, as a regulator of Trk1’s capacity to import potassium. As with internal potassium, we detected a substantial correlation between the increase in affinity and the maximum velocity of Trk1 and the relative hyperpolarization (Figure 1H and Table 1). The increase in membrane potential was not a mere consequence of potassium loss, but rather the regulatory force, as evidenced by the data obtained for the BYT2[YCp352] strain. Although this strain lost a comparable amount of potassium to BYT12[pScTRK1] (approx. 80%, Figure 3B) upon potassium starvation, it was, at the same time, significantly less hyperpolarized (Figure 3C, Table 2) and the increase in affinity of its Trk1 was much less pronounced than in the BYT12[pScTRK1] strain (approx. 5-fold compared to more than 50-fold, Table 2). The correlation between the affinity and membrane potential seems to be the only common pattern of regulation of Trk1’s affinity among the four compared strains. As summarized in Table 2, the increase in affinity closely correlates with the degree of relative hyperpolarization upon potassium starvation. Thus, the dominant signal for increasing the affinity seems to be the level of plasma-membrane hyperpolarization.

The role of membrane potential in the Trk1’s affinity confirms the possibility of a direct functional connection between Pma1 and Trk1. The interdependence of the export of protons and import of potassium on the whole-cell level has been known for a number of years [20,21,22], as well as the mutual dependence of Pma1 and Trk1 transport activities, e.g., reference [23]. Since Pma1 is the main creator of membrane potential in yeast cells, and its increased activity leads to the hyperpolarization of their plasma membranes, it is likely that Trk1 reacts directly to an increase in membrane potential, by increasing its capacity to import potassium, thus, balancing the export of protons. The elevated uptake of potassium could, at the same time, lead to a partial depolarization of the plasma membrane, thus, creating a negative feedback loop that would result in a gradual decrease in the uptake capacity of Trk1.

In the last part of our work, we focused on the putative structural changes that Trk1 undergoes during switching to the high-affinity state. As was mentioned in the Introduction, substitution L949P is the only mutation known to abolish Trk1’s switch to the high-affinity state [17]. In accordance with the published information, our cells expressing the L949P version of Trk1 were specifically susceptible to a very low external concentration of potassium (Figure 4B), and exhibited a severe impairment in switching to the high-affinity state (Figure 4E). To distinguish between the effects of the absence of leucine in position 949 and the introduction of proline, we constructed several additional Trk1 versions. The mutations L949A and L949S had almost no effect on cell growth and kinetic parameters (Figure 4B,E for L949A). These data led us to believe that the effects of the L949P mutation are caused by the introduction of proline, rather than the absence of leucine. Prolines induce distortion in α-helices, as their cyclic side chains introduce a local break (Pro kink). Thus, a prolyl residue introduces a flexible point in the P-helix, which seems to be of both structural and functional importance. The Leu949 residue is located within the P-helix of the third MPM Trk1 domain. The P-helices of the four MPM domains are thought to have a substantial effect on the conformation of the pore and selectivity filter in models of Trk1, other SKT proteins and the KcsA channel [11,12,42]. The introduction of proline into this short helix in Trk1 might potentially lead to a disruption of the conformation of the helix, and consequently to a disruption of the entire pore region. It is, however, striking how this putative local structural change only affects Trk1’s performance in the high-affinity state and has little to no effect on the low-affinity state (Figure 4E). This discrepancy strongly suggests that the entire region, presumably affected by this substitution, might be in a substantially different conformation in the low- and high-affinity states. It is also interesting that this mutation has no effect on the growth of cells on the plates supplemented with high concentrations of lithium and sodium (Figure 4D) and, therefore, probably no effect on the selectivity of Trk1.

The replacement of Leu949 with charged amino-acid residues also gave interesting results. The introduction of a negative charge led to a decrease in affinity in the low-affinity state, but also surprisingly to an increase in affinity and decrease in V_max_ in the high-affinity state, and to diminished growth in the presence of a high external Li^+^/K^+^ ratio, compared to cells with native Trk1 (Figure 4D,E). The lower K_T_ upon potassium starvation can be explained by an elevated local concentration of potassium, due to the presence of a negatively charged glutamic acid in proximity to the pore, and the decrease in V_max_ by a stronger bond between the substrate and high-affinity binding site, similarly described for Nrt1.1 (cf. above and [33]). There could be a similar explanation for the diminished growth upon a high external concentration of lithium. The elevated local concentration increases the chance of lithium being imported non-specifically through Trk1. It is, however, unclear why we have not detected a similar effect for a high external concentration of sodium. The opposite effects of glutamic acid at position 949 on affinity in the low- and high-affinity states again point to the possibility of this region adopting different conformations when in one of the states, as opposed to the other. The negative effect on affinity in the low-affinity state might possibly be caused by the structural impairment of the pore region in this conformational state.

The replacement of Leu949 with positively charged arginine led to significantly diminished growth under all the tested conditions, accompanied by a decrease in affinity in both states. The presence of a positive charge in proximity to the pore region might create an electrostatic barrier to the entrance of potassium ions. Moreover, the observed mislocalization of the L949R version suggests that this mutation also prevents the proper folding and targeting of Trk1 to the plasma membrane (Figure 4C).

When similar mutations were introduced to the other three P-helices (Figure 5), the substitutions L81P and L1115P led to the almost complete abolishment of Trk1’s functionality (Figure 5C), most likely as a consequence of a severe mislocalization of these Trk1 versions (Figure 5D). A number of putative interactions between the amino-acid residues located within the first and fourth MPM domains exist according to the Trk1 model, some of them in the vicinity of L81 and L1115 residues [11]. It is likely that these interactions are disrupted upon the introduction of the proline, and consequently the Trk1 protein is not properly folded. On the other hand, we detected effects similar to the L949P substitution for the F820P substitution in the second MPM domain (Figure 5)—diminished growth on low potassium, an incomplete switch to a high-affinity state and proper localization. As no substantial differences were observed in potassium content or relative membrane potential between the cells expressing the two mutants and the native Trk1 (Figure 6), the signal for a proper affinity switch is similar in all of them, and the observed incomplete switch of the mutated variants to the high-affinity state is most likely a consequence of the structural changes to Trk1.

## 5. Conclusions

In conclusion, our study disproved the concept of a dual-affinity mode of Trk1, and demonstrated that *S. cerevisiae* cells react to potassium conditions by a rapid, continuous and precise adjustment of both the affinity and maximum velocity of their Trk1 proteins. Additionally, we identified a novel important player that participates in the regulation of Trk1’s affinity, membrane potential. The characterization of Trk1 variants with specific mutations suggested structural changes in its pore region, during the transition to the high-affinity state. As the uptake of potassium via Trk1 is involved in yeast cell tolerance to various stresses, e.g., ethanol [43], acidic pH [44,45] or high ammonium [46], and in the virulence of pathogenic yeasts [47], a thorough description of the mechanism and regulation of the changes in Trk1’s uptake capacity is an important step towards understanding the complex mechanisms underlying yeast performance in various biotechnological (often adverse) processes, and also towards the development of new antifungal drugs.

## Figures and Tables

**Table 1:** Correlations among external K^+^ concentration, intracellular K^+^ content, membrane potential and kinetic parameters.

External K^+^	Intracellular K^+^	λ_max_	K_T_	V_max_
[mM]	[nmoles mg^−1^]	[nm]	[µM]	[nmoles mg^−1^ min^−1^]
0.015	330 ± 9	574.43	288.7	51.6
0.05	339 ± 12	574.59	290.8	51.0
0.1	363 ± 3	574.33	287.9	50.5
0.2	418 ± 11	574.05	309.4	44.8
0.3	492 ± 32	573.61	317.6	42.9
0.5	589 ± 26	572.94	328.4	36.9
0.75	720 ± 5	571.95	407.3	30.4
1	796 ± 39	571.22	547.6	25.7
2	824 ± 19	571.03	860.1	26.1
10	919 ± 2	570.92	1590.6	19.7
100	958 ± 41	570.85	1439.4	18.1

Summary of intracellular potassium concentrations, relative membrane potential (λ_max_ from curves in Figure 1H), and rough estimates of kinetic parameters K_T_ and V_max_ of Rb^+^ uptake, obtained for cells incubated at external potassium concentrations, ranging from 15 µM to 100 mM.

**Table 2:** Correlation of changes in kinetic parameters and relative membrane potential upon 1 h of K^+^ starvation.

	**K_m_ [µM]**		
	**NS**	**ST**	**K_T_^NS^/K_T_^ST^**
BYT12 [pCScTRK1]	2488.0	181.1	13.7
BYT12 [pScTRK1]	1968.3	38.2	51.5
BY4741 [YCp352]	1045.7	210.9	5.0
BYT2 [YCp352]	1069.0	203.7	5.2
	**λ_max_ [nm]**		
	**NS**	**ST**	**∆λ_max_^ST-NS^ [nm]**
BYT12 [pCScTRK1]	571.73	574.74	3.01
BYT12 [pScTRK1]	571.89	576.22	4.33
BY4741 [YCp352]	570.21	571.36	1.15
BYT2 [YCp352]	571.27	572.35	1.08

## Data Availability

Not applicable.

## Supplementary Materials

The following supporting information can be downloaded at: https://www.mdpi.com/article/10.3390/jof8050432/s1. Table S1: list of yeast strains; Table S2: list of plasmids; Table S3: list of used oligonucleotides; Figure S1: schematic representation of the predicted transmembrane topology of *Sc*Trk1; Figure S2: localization of GFP-tagged Trk1. References [48,49,50] are cited in the Supplementary Materials.
