# Supplementary material for: Yeast Trk1 Potassium Transporter Gradually Changes Its Affinity in Response to Both External and Internal Signals
Source: J Fungi (Basel). 2022 Apr 22;8(5):432. doi: 10.3390/jof8050432 (PMC9144525; doi:10.3390/jof8050432)
Supplement: Supplementary file 1 [file jof-08-00432-s001.zip › jof-1673309-supplementary.pdf]

Yeast Trk1 potassium transporter gradually changes its affinity in response to both external and internal signals

Jakub Masaryk, Hana Sychrová*

Laboratory of Membrane Transport, Institute of Physiology, Czech Academy of Sciences, Prague, Czech Republic

Supplementary material

Table S1. List of yeast strains

Name	Genotype	Reference
**BY4741**	*MATa his3Δ1 leu2Δ0 met15Δ0 ura3Δ0*	EUROSCARF
**BYT2**	*trk2Δ::loxP*, derived from BY4741	[25]
**BYT12**	*trk1Δ::loxP trk2Δ::loxP*, derived from BY4741	[48]

[25] Petrezselyova, S.; Ramos, J.; Sychrova, H. Trk2 transporter is a relevant player in K^+ supply and plasma-membrane potential control in *Saccharomyces cerevisiae*. *Folia Microbiol*. **2011**, *56*, 23-28.

[48] Petrezselyova, S.; Zahradka, J.; Sychrova, H. *Saccharomyces cerevisiae* BY4741 and W303-1A laboratory strains differ in salt tolerance. *Fungal Biol.* **2010**, *114,* 144-150.

Table S2. List of plasmids

Plasmid	Relevant features	Reference
Empty multicopy plasmids		
pRS316	*URA3*, CEN6ARS4	[49]
YEp352	*URA3*, 2µ origin	[50]
Empty centromeric plasmid		
YCp352	YEp352-CEN6ARS4	This study
	URA3,	
*Multicopy plasmids with TRK1**		
pScTRK1	*ScTRK1* in YEp352	Papouskova (unpublished)
pGRU1ScTRK1	GFP-tagged *ScTRK1*	[24]
	URA3, 2µ origin	
*Centromeric plasmids with TRK1**		
pCScTRK1	pScTRK1-CEN6ARS4	This study
pCScTRK1-GFP	pGRU1ScTRK1-CEN6ARS4	This study
*Centromeric plasmids pCScTRK1 and pCSTRK1-GFP bearing indicated mutations in TRK1**		
pCScTRK1_L949P		This study
pCScTRK1_L949A		This study
pCScTRK1_L949E		This study
pCScTRK1_L949R		This study
pCScTRK1_L949S		This study
pCScTRK1_L81P		This study
pCScTRK1_F820P		This study
pCScTRK1_L1115P		This study
pCScTRK1-GFP_L949P		This study
pCScTRK1-GFP_L949A		This study
pCScTRK1-GFP_L949E		This study
pCScTRK1-GFP_L949R		This study
pCScTRK1-GFP_L81P		This study
pCScTRK1-GFP_F820P		This study
pCScTRK1-GFP_L1115P		This study

**TRK1* expressed under the control of weak and constitutive *S. cerevisiae NHA1* promoter

[49] Sikorski, R.; Hieter, P. A system of shuttle vectors and yeast host strains designed for efficient manipulation of DNA in *Saccharomyces cerevisiae*. *Genetics* **1989**, *122*, 19-27.

[50] Hill, J.; Myers, A.; Koerner, T.; Tzagoloff, A. Yeast/*E. coli* shuttle vectors with multiple unique restriction sites. *Yeast* **1986**, *2*, 163-167.

[24] Zimmermannova, O.; Felcmanova, K.; Rosas-Santiago, P.; Papouskova, K.; Pantoja, O.; Sychrova, H., Erv14 cargo receptor participates in regulation of plasma-membrane potential, intracellular pH and potassium homeostasis via its interaction with K^+-specific transporters Trk1 and Tok1. *Biochim. Biophys. Acta* **2019,** *1866,* 1376-1388.

Table S3. List of used oligonucleotides

Oligonucleotide	Sequence (5'-3')
**2µ-CEN6ARS4 exchange**	
2µ-CEN6ARS4_for	cggcatcagagcagattgtactgagagtgcaccataacgcGGGTCCTTTTCATCACGTGC
2µ-CEN6ARS4_rev	tgctccttccttcgttcttccttctgttcggagattaccgCTTAGGACGGATCGCTTGCC
URA3_for	GTATATTCTCCAGTAGATAGG
CEN6ARS4_rev	GTAAGTTACAGGCAAGCGATCCG
**Site-directed mutagenesis**	
ScTRK1_L949P_for	GCTATAGAGTCCTTGTCGGCCCGTTTCAATCTGTTAGCACAAG
ScTRK1_L949P_rev	CTTGTGCTAACAGATTGAAACGGGCCGACAAGGACTCTATAGC
ScTRK1_L949A_for	GCTATAGAGTCCTTGTCGGCGCGTTTCAATCTGTTAGCACAAG
ScTRK1_L949A_rev	CTTGTGCTAACAGATTGAAACGCGCCGACAAGGACTCTATAGC
ScTRK1_L949E_for	GCTATAGAGTCCTTGTCGGCGAGTTTCAATCTGTTAGCACAAG
ScTRK1_L949E_rev	CTTGTGCTAACAGATTGAAACTCGCCGACAAGGACTCTATAGC
ScTRK1_L949R_for	GCTATAGAGTCCTTGTCGGCCGGTTTCAATCTGTTAGCACAAG
ScTRK1_L949R_rev	CTTGTGCTAACAGATTGAAACCGGCCGACAAGGACTCTATAGC
ScTRK1_L949S_for	GCTATAGAGTCCTTGTCGGCTCGTTTCAATCTGTTAGCACAAG
ScTRK1_L949S_rev	CTTGTGCTAACAGATTGAAACGAGCCGACAAGGACTCTATAGC
ScTRK1_L81P_for	GATACATTGATACACCGTTTTTAGCAGCGGG
ScTRK1_L81P_rev	CCCGCTGCTAAAAACGGTGTATCAATGTATC
ScTRK1_F820P_for	CTACATGGTGGGGACCTTGGACAGCAATGAG
ScTRK1_F820P_rev	CTCATTGCTGTCCAAGGTCCCCACCATGTAG
ScTRK1_L1115P_for	CAAACTTTAATATATTTGCAATTCCTTTTGAAATTGTTAGCGCTTACGG
ScTRK1_L1115P_rev	CCGTAAGCGCTAACAATTTCAAAAGGAATTGCAAATATATTAAAGTTTG

*underlined: codon for substituting amino acid

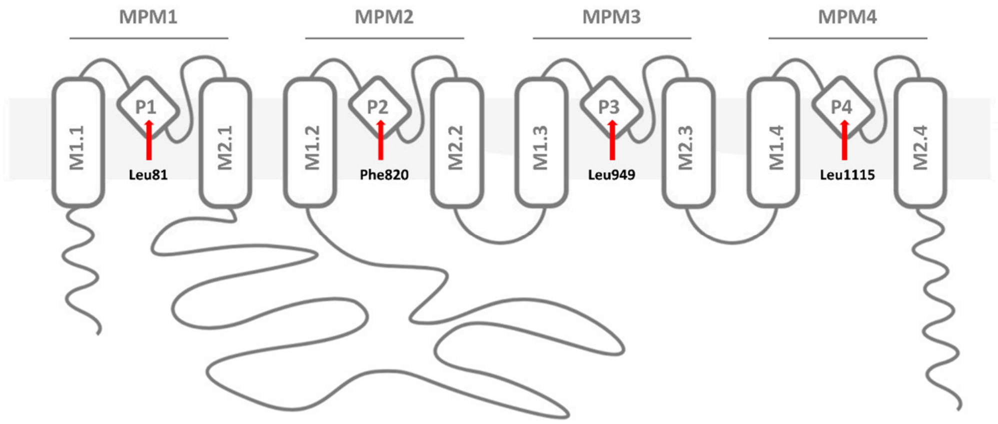

Figure S1: Schematic representation of the predicted transmembrane topology of *ScTRK1*. Highlighted are positions of residues studied in this work.

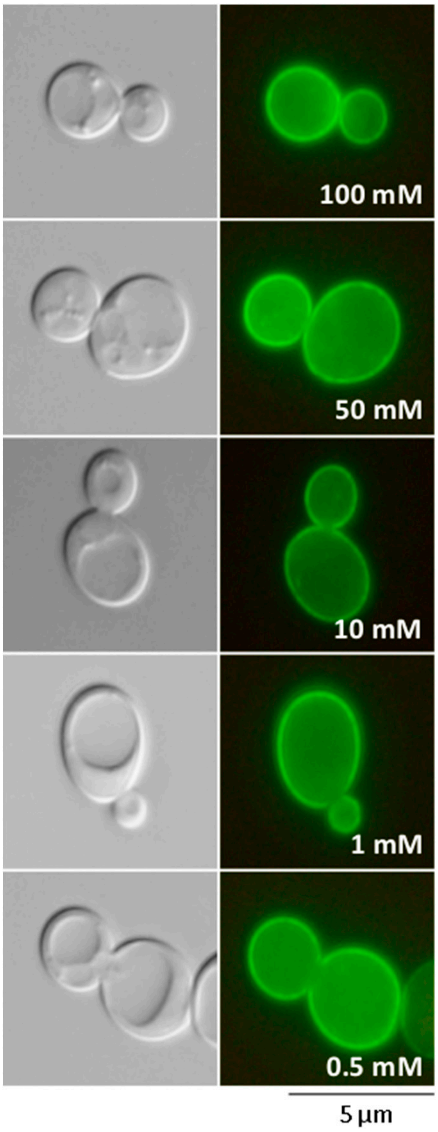

Figure S1. Localization of GFP-tagged Trk1. Cells were grown in YNB-F supplemented with indicated KCl concentrations to $OD_{600} \sim 1$, and visualized using Olympus Bx53 microscope and captured by Olympus DP73 camera.
